# Summer Diarrheas of Children and Their Treatment

**Published:** 1902-08

**Authors:** M. A. Auerbach

**Affiliations:** New York City. Medical Inspector, Department of Health


### Summer Diarrheas of Children and Their Treatment.

By M. A. AUERBACH, Ph. G., M. D., New York City.
Medical Inspector, Department of Health.

THE importance of these demand a separate consideration. Three forms, more or less distinct, can be recognised—namely, acute dyspeptic diarrhea, cholera infantum, and acute enterocolitis.

*Acute Dyspeptic Diarrhea.*—This disease is chiefly due to errors in diet, which do not necessarily consist in the substitution of unnatural foods for the mother's milk. The mother's milk may be altered in quality by emotional causes, by improper food and improper hygiene, or it may be caused by over-frequent nursing. More often, however, it is caused by the ingestion of unnatural foods.

There are also predisposing influences which facilitate the action of the exciting causes. These are especially dentition and the extreme heat of summer. The prognosis of the disease among the better classes is commonly favorable, but among the weak, puny, and half-starved children of our lower east side, large numbers perish, especially during the summer months.

The old time treatment in these cases was a primary purge with calcined magnesia or castor oil. After the purge bismuth subnitrate or prepared chalk was given. Since the introduction of glyco-thymoline, the above mentioned methods have been cast aside. A very good and effective prescription which has given me most splendid results in these kind of cases, in conjunction with a carefully restricted diet is:

| | | |
|---|---|---|
| ℞ | Bismuth subnitratis . . . . . . . . . . . . . . . . . . . . | ʒi. |
| | Tr. opii deoderatum . . . . . . . . . . . . . . . . . . . . | m. x. |
| | Glyco-thymoline . . . . . . . . . . . . . . . . . . . . | ℥ii. |
| | Aqua rosarum ad. q. s . . . . . . . . . . . . . . . . . . . | ℥iv. |
| | Misce et | |

Sig. dr. 1 every 3 hours. (For a child one year of age.)

*Cholera Infantum* is a variety of acute catarrhal enteritis of intense severity, corresponding in symptoms and course to cholera morbus in the adult, but much more serious in termination. The prognosis in these cases is at best not very favorable, although recovery is not impossible.

The treatment is of quite a different nature from that above mentioned. In the first place, the fever must be combated, and I know of no better method than a bath containing some glyco-thymoline, at about 80° F., reduced by adding small pieces of ice to 70° or 65°. Next, pain should be controlled by 1-100 of a grain of morphine sulphate, administered to a child of one year; then stimulation with strychnia, hypodermatically, iced champagne to prevent vomiting, brandy, whiskey and other stimulants.

One of the best methods for irrigating the large intestine is by introducing a small, soft catheter through the rectum and injecting into the bowel about a pint to a pint and a half of warm water, containing about 25 per cent. of glyco-thymoline. This I find removes and prevents the reaccumulation of the fermentive as well as the putrefactive products of the bowel. Should, however, the hyperpyrexia continue, the douche may be given at a lower temperature. During convalescence great care must be taken in feeding of the patient.

*Acute Enterocolitis* is an affection of an inflammatory nature, more severe than dyspeptic enteritis, chiefly of the ileum and colon, affecting especially the lymph follicles. This like the preceding is a disease of the hot months of summer, and especially the period of teething. It is produced by the same causes as dyspeptic diarrhea. It is most frequent between the ages of 6 and 18 months. It likewise may be a termination of dyspeptic diarrhea, or of cholera infantum.

In the treatment the general surroundings and hygiene necessarily play an important part. The medical treatment, however, is somewhat different from the other. Anodynes are most imperatively demanded because there is greater suffering, and depletion may be needed in the beginning by salines, though good judgment is required because the child's strength must be watched. The colon should be flushed with a solution of glyco-thymoline having a strength of 25 per cent. This I find answers admirably in these cases. The solution may be made with iced water. The coming teeth should likewise be watched and the gums be scarified whenever required.

I will supplement my remarks by adding a few of the many cases treated with glyco-thymoline and leave results to speak for themselves.

Case I.—M.K., aged 8 months; male; was taken with severe vomiting and colicky pains at night. The vomita contained lumps of coagulated milk. The stools were very offensive and recurred at intervals of twenty minutes. I left a prescription for glyco-thymoline, 2 oz., bismuth subnitrate, 1 dr., rose water enough to make 4 ozs. Next morning, finding but little improvement in my patient, the bowel was at once flushed out with a 25 per cent. aqueous solution of glyco-thymoline, and the prescription given the night previous was continued. This treatment was maintained for three days, the patient steadily improving during that time. I recommended that the child be taken away from the city, which was done. The parent reported later that the child had not had a relapse but made a speedy recovery.

Case II.—Mary C., age 7½ months was brought to my office with her knees drawn up, a look of anguish on her face, which was pale and drawn, with eyes protruding. She had a number of watery discharges from the bowels, incessant vomiting, a temperature of 103½°, a rapid and feeble heart. A further examination was unnecessary. Anodynes were at once administered, to soothe the pain. I washed out the bowels with a 40 per cent. solution of glyco-thymoline, and administered the same in a 50 per cent. solution, with peppermint water internally, in doses of 1 teaspoonful, repeated every two hours. The results in this case far exceeded my expectations The child made a slow but successful recovery.

Case III.—L.P., male child, age 16 months; was called to check the diarrhea, which was of a severe character having a tinge of blood. The vomiting was not of a serious nature; the only alarming symptoms being intestinal. Three separate washings of the child's colon were made at intervals of six hours. Food was restricted to barley water; this case, like the one preceding, made a perfect and speedy recovery.

Case IV.—R. A., a little tot of the east side, aged 13 months, brought up in one of the dark and dingy rooms of a tenement house; this poor little one was suffering for five days before my attention was called to the case. I found it in an emaciated condition, unable to move a limb, the bowel movements were frequent and watery; collapse was imminent. Strychnia was administered, hypodermatically, to stimulate the heart, after which diluted brandy was given every half hour. The colon was irrigated with 24 oz. of a 50 per cent. aqueous solution of glyco-thymoline. I had the child under my observation for two and one half weeks, and with proper food and fresh air, a good recovery resulted.

